# Ectopic Pregnancy With Low Beta-Human Chorionic Gonadotropin (HCG) Managed With Methotrexate and Progressed to Rupture

**DOI:** 10.7759/cureus.18749

**Published:** 2021-10-13

**Authors:** Madhumitha Prabhakaran, Anju Beesetty

**Affiliations:** 1 Obstetrics and Gynecology, Saveetha Medical College and Hospital, Chennai, IND; 2 Obstetrics and Gynecology, Southern Medical University, Guangzhou, CHN

**Keywords:** ectopic pregnancy, treatment protocol, acute pain in pregnancy, ob-gyn, patient under observation, high dose methotrexate, surgical evacuation, ruptured tubal ectopic pregnancy, ruptured ectopic pregnancy

## Abstract

Ectopic pregnancies are one of the most common obstetric diagnoses made in the emergency department. Once diagnosed, patients can be managed expectantly, medically, or surgically. Decisions regarding patient management are made using evidence-based protocols. Hemodynamically stable patients with reduced beta-human chorionic gonadotropin (HCG) levels and a small mass on ultrasonography are managed with methotrexate therapy. Although most patients adequately treated with methotrexate resolve, there are a few rare instances where patients progress to develop a ruptured ectopic despite having low and declining beta-HCG levels. These patients must be taken for surgical evacuation at the earliest opportunity to prevent life-threatening hemorrhage. Hence, obstetricians must be prepared for such potential complications of low-risk ectopic pregnancies.

## Introduction

An ectopic pregnancy is one in which the fertilized egg implants at any site other than the endometrium of the uterine cavity. Most of the time, implantation of the blastocyst occurs in the fallopian tubes, but it can also occur in the abdomen, ovary, or cervix. Patients commonly present with pelvic pain, vaginal bleeding, and amenorrhea. Risk factors include scarring to the fallopian tubes as a result of tubal surgery, pelvic inflammatory disease, endometriosis, or prior ectopic pregnancy. Conditions that reduce tubal motility like the use of progesterone-only pills, smoking, and Kartaneger's syndrome also increase the risk of implantation in the fallopian tubes [1]. All cases of ectopic pregnancies, regardless of location, are an obstetric emergency. Life-threatening complications include tubal rupture and hemoperitoneum. Ectopic pregnancies account for up to 10% of all maternal deaths. Furthermore, up to 75% of deaths from ectopic pregnancies are considered preventable, indicating the importance of making a prompt diagnosis and starting appropriate treatment [2].

## Case presentation

The patient was a 37-year-old woman (gravida 6, para 4, abortion 1, live 4) who presented to the emergency department with lower back pain, cramping pelvic pain, and spotting. Her last period was one month ago, and she had a positive home pregnancy test one week ago. She had no previous medical history and was not taking any medications. Ultrasound showed no evidence of an intrauterine gestational sac. However, there was a 1.3 cm x 1.1 cm x 1.1 cm complex lesion medial to the right ovary with a gestational sac-like structure and echogenic rim. There was no free fluid. A diagnosis of unruptured right adnexal ectopic pregnancy was made. Beta-HCG levels were found to be elevated to 454.1 mIU/mL and methotrexate therapy was started. Monitoring on Day 4 showed a beta-HCG level of 635.8 mIU/mL. The patient was administered a second dose of methotrexate under observation, after which she experienced abdominal pain and dizziness. Repeat ultrasound showed a 1.1 cm cystic mass adjacent to the right ovary with a small amount of free fluid. The patient remained hemodynamically stable. The decision was made to surgically manage the patient due to the rising beta-HCG levels and the clinical symptoms. Intraoperative findings showed a right tubal ampullary pregnancy 4 cm x 4 cm in size, with the ovary intact and 30-50 cc of hemoperitoneum. The pathology report verified the same. The postoperative period was uneventful, and the patient recovered well with beta-HCG levels returning to the baseline.

**Table 1:** Serial beta-HCG monitoring HCG: human chorionic gonadotropin

DAY	BETA-HCG LEVEL
0	454.1 mIU/mL
4	635.8 mIU/mL
7	522.3 mIU/mL
14	144 mIU/mL
21	undetectable

## Discussion

Ectopic pregnancies are obstetric emergencies that require immediate diagnosis and appropriate treatment. The decision has to be made between following expectant, medical, or surgical management. In this patient, after taking the beta-HCG levels, pelvic ultrasound findings, and clinical presentation into account, we opted to start medical management with methotrexate. According to the American College of Obstetricians and Gynecologists (ACOG) Practice Bulletin 191, patients who are candidates for medical therapy should be hemodynamically stable, have an unruptured mass, and have no absolute contraindications to methotrexate [1]. According to the American Academy of Family Physicians (AAFP), expectant management can be considered when the patient is hemodynamically stable, the beta-HCG level is less than 1500 mIU/mL and fails to double in 48 hours, and the patient is reliable for follow-up. If not, medical management with methotrexate can be considered. Contraindications to methotrexate therapy include a significant risk associated with general anesthesia, lack of patient compliance, fetal cardiac motion, hemodynamic instability, intrauterine pregnancy, and an ectopic mass greater than 3.5 cm in the largest dimension. If there is any contraindication to medical therapy, the patient should be surgically managed. According to these guidelines, the patient under discussion is eligible for medical therapy and has no contraindications for methotrexate.

According to a study done by Tenore JL, when the pretreatment beta-HCG levels are less than 5,000 mIU/ml, the failure rate is only 3.7% as compared to a failure rate of 14.3% when the pretreatment beta-HCG levels are more than 5,000 mIU/mL [3]. Similarly, a study done by Corsan et al. found that there is a higher risk of treatment failure only when the beta-HCG levels are greater than 1,500 mIU/mL [4]. This patient had a pretreatment beta-HCG level of 454.1 mIU/mL. Unfortunately, after the second dose, the patient showed clinical features suggestive of a ruptured ectopic and was promptly taken for surgical evacuation. This raises the important question of when to avoid medical management and proceed to surgical management despite low and declining beta-HCG levels.

In a study done by Saxon et al. of 716 patients admitted with ectopic pregnancy, 29% of those with a beta-HCG level of less than 100 mIU/mL were found to have tubal rupture during laparoscopy [5]. From this, we can conclude that the risk of tubal rupture varies across a wide range of beta-HCG levels and is therefore not always a reliable indicator of deferring surgical management for medical management.

## Conclusions

Ectopic pregnancies that are not adequately managed have a high mortality rate. The most common complication is rupture of the ectopic, which leads to hemorrhage, hemodynamic instability, and eventually death. The high mortality rates of the situation emphasize the importance of careful management of ectopic pregnancies. Although a low serum beta-HCG level has been reassuring in the past for a relatively benign course; this is not always the case. There is always a risk of medical treatment failure and rupture that physicians must look out for.
